# Acute and chronic impact of cardiovascular events on health state utilities

**DOI:** 10.1186/s12913-015-0772-9

**Published:** 2015-04-22

**Authors:** Louis S Matza, Katie D Stewart, Shravanthi R Gandra, Philip R Delio, Brett E Fenster, Evan W Davies, Jessica B Jordan, Mickael Lothgren, David H Feeny

**Affiliations:** 1 Outcomes Research, Evidera, 7101 Wisconsin Avenue, Suite 1400, Bethesda, MD USA; 2 Global Health Economics, Amgen Inc, One Amgen Center Drive, Thousand Oaks, CA USA; 3 Neurology Associates of Santa Barbara, 219 Nogales Avenue, Suite F, Santa Barbara, CA USA; 4 Division of Cardiology, National Jewish Health, 1400 Jackson Street, Denver, CO USA; 5 Outcomes Research, Evidera, Metro Building, 6th Floor, No. 1 Butterwick, London, UK; 6 Global Health Economics, Amgen (Europe), Dammstrasse 23, P.O. Box 1557, CH-6301 Zug, Switzerland; 7 Department of Economics, McMaster University, KTH 426 Hamilton, ON Canada

**Keywords:** Utility, Cardiovascular disease, Acute coronary syndrome, Heart failure, Stroke, Cost-utility, Time trade-off

## Abstract

**Background:**

Cost-utility models are frequently used to compare treatments intended to prevent or delay the onset of cardiovascular events. Most published utilities represent post-event health states without incorporating the disutility of the event or reporting the time between the event and utility assessment. Therefore, this study estimated health state utilities representing cardiovascular conditions while distinguishing between acute impact including the cardiovascular event and the chronic post-event impact.

**Methods:**

Health states were drafted and refined based on literature review, clinician interviews, and a pilot study. Three cardiovascular conditions were described: stroke, acute coronary syndrome (ACS), and heart failure. One-year acute health states represented the event and its immediate impact, and post-event health states represented chronic impact. UK general population respondents valued the health states in time trade-off tasks with time horizons of one year for acute states and ten years for chronic states.

**Results:**

A total of 200 participants completed interviews (55% female; mean age = 46.6 y). Among acute health states, stroke had the lowest utility (0.33), followed by heart failure (0.60) and ACS (0.67). Utility scores for chronic health states followed the same pattern: stroke (0.52), heart failure (0.57), and ACS (0.82). For stroke and ACS, acute utilities were significantly lower than chronic post-event utilities (difference = 0.20 and 0.15, respectively; both p < 0.0001).

**Conclusions:**

Results add to previously published utilities for cardiovascular events by distinguishing between chronic post-event health states and acute health states that include the event and its immediate impact. Findings suggest that acute versus chronic impact should be considered when selecting scores for use in cost-utility models. Thus, the current utilities provide a unique option that may be used to represent the acute and chronic impact of cardiovascular conditions in economic models comparing treatments that may delay or prevent the onset of cardiovascular events.

**Electronic supplementary material:**

The online version of this article (doi:10.1186/s12913-015-0772-9) contains supplementary material, which is available to authorized users.

## Background

Cardiovascular disease is a worldwide health concern and the primary cause of non-communicable disease-related death in the United States and Europe [1-4]. Cardiovascular conditions such as heart disease and stroke are associated with serious acute symptoms [5-8], long-term disability [9-11], and substantial costs for patients and healthcare systems [1,12,13]. Treatments such as statins have been shown to reduce the risk of cardiovascular events [14-16], and new treatments are in development [17-19].

As new treatments are introduced, cost-effectiveness analyses are needed to examine and compare their value relative to existing treatments. Cost-utility analyses (CUA) are a type of cost-effectiveness analysis commonly conducted to examine treatments for cardiovascular disease and risk factors such as hyperlipidemia [20-23]. A CUA requires health state utility values, which represent the strength of preferences for various health states and are used to calculate quality-adjusted life years (QALYs) [24-26]. A recent systematic review found that a wide range of published utility values are available to represent health states with cardiovascular conditions such as stroke, acute coronary syndrome (ACS), and heart failure [27].

An important limitation of available cardiovascular utilities is that the time between the cardiovascular event and the utility assessment is not usually reported. For example, commonly cited studies have derived utilities from large surveys in which individuals provided assessments of their own health using a generic preference-based measure [28,29]. These studies reported utilities for subgroups of patients who had experienced cardiovascular events, such as stroke or myocardial infarction, at an unspecified time in their medical history. Therefore, the resulting utility values represent a post-event health state rather than the event itself, and the utility assessment may have occurred many years after the cardiovascular event when patients may have substantially recovered. Because time between the cardiovascular event and utility assessment is not known, the utilities do not necessarily capture the impact of the event or the substantial quality-of-life impact that occurs during the first months or even years following the event. Due to this limitation, utilities from studies that do not report duration of time between the cardiovascular event and utility assessment are difficult to interpret and may underestimate the disutility of cardiovascular events.

Some studies address this problem by documenting and reporting the time between the cardiovascular event and utility assessment. For example, studies conducted in Thailand, the Netherlands, Sweden, and the UK have reported EQ-5D values for stroke patients at specific time points after the event including two months [30], three months [31], six months [30-32], nine months [31], and/or 12 months [31] post-stroke. Other studies have evaluated utilities at specific time points in relation to hospital discharge [32-36]. Studies in the UK, Australia, and multiple European countries have identified EQ-5D values for myocardial infarction or angina patients before hospital discharge [37], at discharge [38], six weeks [37,39], three months [37], and/or one year [38] after the event. However, all of these studies may also be underestimating the impact of cardiovascular events because they do not capture the disutility of the event itself.

Therefore, the purpose of this study was to estimate utilities of health states representing cardiovascular conditions while distinguishing between chronic post-event impact and acute impact including the cardiovascular event. Researchers have commented that it would be difficult or perhaps impossible to have patients complete a generic preference-based measure (e.g., EQ-5D) or another type of utility rating in the acute phases immediately following a major cardiovascular event, such as a stroke [30,40-42]. As an alternative method, the current study used a health state description (often called a vignette-based) utility assessment approach, which allowed for assessment of chronic post-event health states as well as acute health states including the event itself. Acute health states described a year beginning with the cardiovascular event, followed by description of a typical course of recovery for the remainder of the one-year duration. Chronic health states described patients experiencing ongoing effects of cardiovascular events that occurred in the past. All health states were valued by general population respondents in time trade-off (TTO) tasks, with a one-year time horizon for acute health states and a 10-year time horizon for chronic health states.

## Methods

### Overview of study design

Health state descriptions were drafted based on literature review and input from clinicians who treat cardiovascular disease. Then, the health states were refined based on additional clinician interviews and a pilot study with general population respondents in Edinburgh, UK. Finally, health states were rated in a TTO valuation study with general population participants in Edinburgh and London, UK.

Three cardiovascular conditions were represented in the health states (stroke, ACS, heart failure). For each of these three conditions, a one-year health state was drafted to represent the acute impact, and a post-event health state was drafted to represent ongoing chronic impact. Every participant rated all six health states, and to control for order effects, participants were randomized to begin with either the chronic or the acute health states.

### Health state development

Throughout the health state development process, telephone interviews were conducted with a total of nine clinicians (all with MD and/or MBChB degrees) including six cardiologists, two neurologists (who primarily described patients’ experiences of stroke), and one chemical pathologist specializing in diseases related to hyperlipidemia. Five of the clinicians were from the US, while the other four were based in the UK. In the initial phase, telephone interviews were conducted with six clinicians to inform health state development with questions focusing on patients’ typical experiences with stroke, ACS, and heart failure including symptoms; treatment procedures and regimens; and acute and chronic impact. To characterize the acute health states, clinicians were asked to describe the typical patient experience of the event itself; experiences during and after hospitalization; typical course of recovery; duration of treatment and hospitalization; and impact on functioning during the first year after the event. For chronic health states, clinicians were asked to describe typical ongoing symptoms, treatment, and impact in years following an event. Although there is substantial variation in patients’ experiences of cardiovascular events, clinicians were asked to try to focus on the “most typical patient experience” when providing descriptions.

Draft health states were then sent to two of the original six clinicians and three new clinicians who provided comments regarding the clarity and accuracy of the descriptions. This process of refining the health states was iterative, and several of the clinicians were interviewed multiple times as health states were revised during this second phase of interviews. Clinicians’ descriptions of the health states were generally consistent with each other. For some relatively minor points pertaining to treatment (e.g., frequency of clinic visits, typical duration of hospital stay), there were occasional discrepancies between US and UK clinicians. When these discrepancies arose, health states were drafted to be consistent with treatment patterns reported by UK clinicians because these descriptions were intended to be valued in the UK.

Literature review was conducted throughout the health state development process to inform the clinician interview questions and ensure that the health state descriptions were consistent with published research. Literature searches focused on the symptoms, impact, and treatment of stroke [8,9,14], ACS [5,7,43], and heart failure [15,44,45]. The stroke literature revealed the greatest degree of heterogeneity in patient experience, ranging from mild stroke with complete recovery to severe stroke with long-term impairment in speech, cognition, and mobility [8-10]. During the clinician interviews, the clinicians were asked to provide descriptions of a stroke of moderate severity. Initially, literature searches were conducted to identify distinct characteristics of myocardial infarction and unstable angina. However, clinicians reported that patients’ experiences of these two cardiovascular events and their treatment were quite similar, and published literature often groups these events into the single category of ACS [5-7,46]. Therefore, acute and chronic health states representing ACS were drafted, instead of separate health states representing myocardial infarction and unstable angina.

### Pilot study and selection of utility elicitation methods

After conducting the second round of clinician interviews and making the corresponding health state revisions, the health states were tested in a pilot study with 20 general population participants in Edinburgh, Scotland (13 female; mean age = 41.5 years; age range = 21 to 74) recruited via newspaper and online advertisements. In order to identify the most appropriate utility valuation method for these health states, each participant valued the states using multiple methods including TTO and standard gamble (SG) with several time horizons [24-26]. In utility elicitation procedures, particularly TTO, the duration of time in the health state being rated (i.e., the time horizon) is an important component of the task. This time horizon varies across TTO studies [47]. The most commonly used TTO time horizon appears to be 10 years, which provides for comparability with previously published TTO utility studies including the influential Measurement and Valuation of Health (MVH) study that elicited utilities for EQ-5D health states [48,49]. However, other time horizons are also frequently used, including shorter durations intended to match the typical clinical course of the health state [50-52] or longer time horizons corresponding to each respondent’s additional life expectancy [52-57]. In the pilot study, 1-year and 6-month time horizons were attempted for the acute health states. For the chronic health states, two time horizons were also attempted: a 10-year time horizon and a time horizon based on each respondent’s self-reported life expectancy. The order of the methods (TTO vs. SG) and time horizons was varied.

In this pilot study, participants consistently reported that the health states were clear and easy to understand. Some participants suggested minor revisions in formatting and word choice, and the health states were edited accordingly. All TTO and SG methods yielded utility scores in a reasonable range with logical discrimination among health states. The TTO method was selected for use in the subsequent main study because it was relatively easy for participants to understand and complete, and because it is consistent with the methods used in many recent utility valuation studies, including the MVH study [48,49].

Based on pilot study results, the 1-year time horizon was selected for the acute health states because some participants seemed resistant to trading time in the 6-month TTO, which resulted in a ceiling effect. For chronic health states, the 10-year time horizon was selected to maintain consistency with the MVH study. Furthermore, although the additional life expectancy time horizon yielded logical results, it resulted in implausible clinical situations in some cases. For example, younger respondents with longer additional life expectancies could be presented with an option of living with chronic heart failure for 40 years, which may be unrealistically long for this cardiovascular condition.

### Final health states administered in the time trade-off interviews

Six health states describing cardiovascular events were rated in TTO interviews (see Additional file 1 for full health state text). To avoid the potential bias that could be introduced by health state labels, none of the descriptions provided the name of the cardiovascular condition (i.e., health states did not mention the terms stroke, myocardial infarction, heart attack, ACS, unstable angina, or heart failure) [58]. Instead, the health states described the relevant events, along with symptoms, treatment, and impact of the events.

Three acute health states described a single year in which a cardiovascular event occurred. The acute stroke (health state A) and ACS (B) descriptions consisted of bullet points grouped into four chronologically ordered sections: the event, the hospital experience, the first 6 months after the event, and 7 to 12 months after the event. The acute heart failure health state (C) described a year in which heart failure occurred for a patient who was at risk of this event due to previous ACS. This health state description included: a brief explanation of the event that occurred before the current year (i.e., ACS), current health subsequent to the earlier event, and the event during the current year (i.e., heart failure). This health state structure was designed to reflect clinicians’ descriptions of the most typical patient who experiences acute heart failure.

The three chronic health states described ongoing health of individuals with cardiovascular disease. These health states can be characterized as *post-event health states* that describe the ongoing impact of a previous event on subsequent years, rather than the acute impact of the event itself. All three descriptions briefly mentioned the prior event, while focusing primarily on current ongoing health, treatment, and impact. Health states D and E represented long-term effects of stroke and ACS, respectively. Health state F described a patient with chronic heart failure, following ACS that had occurred in a prior year.

### Participants

Participants were required to be at least 18 years old; able to understand the assessment procedures; able and willing to give written informed consent; and residing in the UK. Inclusion criteria did not specify particular clinical characteristics because interviews were intended to yield utilities that may be used in cost-utility analyses for submission to health technology assessment agencies, most of whom prefer that utilities represent general population values [59-62]. Participants were recruited via advertisements in three newspapers in Edinburgh, two newspapers in London, and the website Gumtree.com. Potentially interested individuals left voicemail messages with their contact information.

### Utility interview procedures and scoring

Utilities were derived by eliciting values for the health state descriptions in a TTO utility interview with a one-year time horizon for acute health states and a 10-year time horizon for chronic health states. All participants evaluated two groups of health states: acute (A, B, C) and chronic (D, E, F). To control for order effects, participants were randomly assigned to rate either the acute or chronic group first, followed by the other group. Prior to beginning TTO procedures, participants completed an introductory ranking task intended to familiarize them with the health state content. During the ranking task, the three health state descriptions (i.e., either acute or chronic) were presented to participants on individual cards. Participants were asked to read the cards carefully and place them in order from most preferable to least preferable. All participants completed the ranking task and the TTO for the first group of three health states before proceeding to the second group of health states.

For TTO ratings of the acute health states (A, B, C), participants were offered a choice between spending one year in the health state being rated or shorter amounts of time in full health (with choices varying in 1-month increments). For TTO ratings of the chronic health states (D, E, F), participants were offered a choice between spending 10 years in the health state being rated or a shorter duration in full health (with choices varying in 1-year increments). Each health state considered better than dead received a utility value on a scale with the anchors of dead (0) and full health (1). The assigned value was calculated based on the choice in which the respondent is indifferent between y months/years in the health state being evaluated and x months/years in full health (followed by dead). The resulting utility estimate (u) is calculated as u = x / y.

If participants indicated that a health state was worse than dead, the interviewer altered the task so that respondents were offered a choice between immediate death (alternative 1) and a 1-year/10-year life span (alternative 2) beginning with varying amounts of time in the health state being rated, followed by full health for the remainder of the time horizon (one example of alternative 2 with a 10-year TTO task would be three years in health state D, followed by 7 years in full health). For these health states, the current study used a bounded scoring approach, which is commonly used for negative utilities [24]. This scoring approach limits the utility range of health states worse than dead to values between 0 and −1. To compute these bounded negative utility values, the Dolan method was used [49], as described by Rowen & Brazier [63]. This method uses the formula u = −x / t, where x is the duration of time in full health and t is the total life span of alternative 2 in the TTO choice. In the current study, t was one year for acute states and 10 years for chronic states, which were the periods of time in the health states being rated plus subsequent months/years in full health.

### Data collection and statistical analysis procedures

Interviews were conducted in private conference rooms in London and Edinburgh in November 2013. All procedures and materials were approved by an independent Institutional Review Board (Ethical & Independent Review Services; Study Number 13105–01), and every participant provided written informed consent before completing any study procedures. Participants completed a brief demographic and clinical form, followed by the TTO utility interview described above. Statistical analyses were completed using SAS version 9.2 (SAS Institute, Cary, NC).

Continuous variables, including utilities and pairwise differences between health state utilities, are summarized in terms of means and standard deviations, and categorical variables such as gender and racial/ethnic background are summarized as frequencies and percentages. Demographic characteristics of the London and Edinburgh subgroups were compared with chi-square analyses (for categorical variables) and t-tests (for continuous variables). Descriptive statistics were also used to summarize willingness to trade time and rates of positive and negative utilities for each health state. In addition, pairwise comparisons were performed using *t*-tests to compare health states within each time horizon (e.g., acute stroke vs. acute ACS) and to compare analogous cardiovascular events across the time horizons (e.g., acute stroke vs. chronic stroke).

## Results

### Sample description

A total of 268 potential participants were reached for screening. Of these, 262 were eligible, 219 were scheduled for interviews, and 206 attended the interviews. Six of the 206 participants were unable to complete the TTO interview procedures to provide valid TTO data. Thus, a total of 200 valid utility interviews were completed.

The sample was 55% female (n = 110), with a mean age of 46.6 years (Table 1). The majority of participants reported ethnicity as white (78.0%), and more participants reported being single (49.0%) than married/living with a partner (36.5%). Most participants reported being employed (28.0% full-time and 33.5% part-time). Less than half of the sample had completed a university degree (n = 89; 44.5%). When asked to report health conditions, the most common responses were depression (n = 29; 14.5%), arthritis (n = 28; 14.0%), diabetes (n = 12; 6.0%), hypertension (n = 19; 9.5%), asthma (n = 9; 4.5%), cancer (n = 9; 4.5%), and thyroid conditions (n = 9; 4.5%). Only four participants reported cardiovascular conditions, two with stable angina (1.0%) and two who had experienced a myocardial infarction (1.0%).

**Table 1:** **Demographic characteristics**

Characteristic	Edinburgh (N = 107)	London (N = 93)	Participants in the analysis sample	p-value^1^
			(N = 200)	
**Age (Mean, SD)**	48.4 (15.5)	44.5 (14.1)	46.6 (14.9)	0.068
**Gender (n, %)**				
Male	52 (48.6%)	38 (40.9%)	90 (45.0%)	0.27
Female	55 (51.4%)	55 (59.1%)	110 (55.0%)	
**Racial/Ethnic Background (n, %)**				
White	100 (93.5%)	56 (60.2%)	156 (78.0%)	<0.0001
Mixed	1 (0.9%)	8 (8.6%)	9 (4.5%)	
Asian	2 (1.9%)	12 (12.9%)	14 (7.0%)	
Black	1 (0.9%)	15 (16.1%)	16 (8.0%)	
Other^2^	3 (2.8%)	2 (2.2%)	5 (2.5%)	
**Marital Status (n, %)**				
Single	44 (41.1%)	54 (58.1%)	98 (49.0%)	0.053
Married/Living with partner^3^	46 (43.0%)	27 (29.0%)	73 (36.5%)	
Other^4^	17 (15.9%)	12 (12.9%)	29 (14.5%)	
**Employment Status (n, %)**				
Full-time work	28 (26.2%)	28 (30.1%)	56 (28.0%)	0.74
Part-time work	34 (31.8%)	33 (35.5%)	67 (33.5%)	
Unemployed	9 (8.4%)	7 (7.5%)	16 (8.0%)	
Other^5^	36 (33.6%)	25 (26.9%)	61 (30.5%)	
**Education Level (n, %)**				
University degree	48 (44.9%)	41 (44.1%)	89 (44.5%)	0.91
No University degree	59 (55.1%)	52 (55.9%)	111 (55.5%)	

^1^P-values are based on t-tests for continuous variables and chi-square analyses for categorical variables.

^2^Other self-reported background includes Arab (n = 3) and Latino (n = 2).

^3^Includes married (n = 64) and living with partner (n = 9).

^4^Includes divorced (n = 20), separated (n = 6), and widowed (n = 3).

^5^Includes retired (n = 29), student (n = 15), homemaker (n = 6), disabled (n = 6), “career break” (n = 1), caregiver for elderly parent (n = 1), “live on investments” (n = 1), volunteer (n = 1), and unspecified (n = 1).

There were no significant differences between the London (n = 93) and Edinburgh (n = 107) samples in age, gender, marital status, or employment status (Table 1). The Edinburgh sample had a significantly higher percentage of white participants than the London sample (93.5% vs. 60.2%; p < 0.0001).

### Health state rankings and utilities

In the introductory ranking task, the acute health states were ranked separately from the chronic health states, and rankings ranged from 1 (most preferable health state) to 3 (least preferable health state). For both acute and chronic health states, stroke was consistently ranked as least preferable. Mean rankings for the acute health states were 1.22 (health state B; ACS), 1.96 (C; acute heart failure), and 2.83 (A; stroke). Mean chronic health state rankings were 1.03 (E; ACS) 2.42 (F; chronic heart failure), and 2.56 (D; stroke).

**Figure 1:**
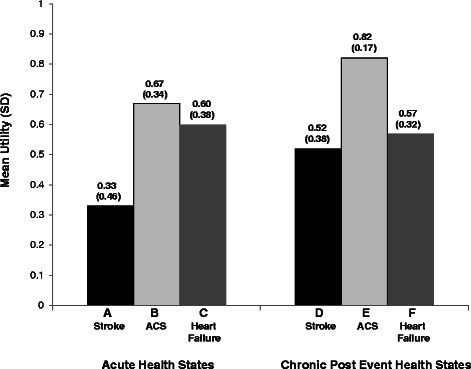
Mean TTO^1^ utility scores (N = 200). ^1^TTO utility scores are on a scale anchored with 0 representing dead and 1 representing full health. TTO = Time trade-off; ACS = Acute coronary syndrome.

Mean health state utility scores followed the same order as the health state rankings (Figure 1). For the acute health states, the cardiovascular event with the lowest utility was stroke (health state A; utility = 0.33), followed by heart failure (C; 0.60) and ACS (B; 0.67). Utility scores for chronic health states followed the same pattern with the lowest utility for stroke (D; 0.52) and higher scores for heart failure (F; 0.57) and ACS (E; 0.82).

### Health state comparisons

Each of the acute health states was significantly different from the other two (Table 2). For example, acute stroke (A) had a significantly lower utility than both acute ACS (B) and heart failure (C) with substantial differences between health states (difference scores = 0.34 and 0.28, respectively; both p < 0.0001). The difference between utilities for ACS and heart failure was also statistically significant (p < 0.0001) but of a smaller magnitude (difference = 0.07).

**Table 2:** **T-tests comparing select pairs of health state utilities (N = 200)**

Comparison	Health state	Mean (SD) utilities^1^	Mean (SD) difference score	t-statistic	p-value
**Acute Health States**					
A vs. B	A. Stroke	0.33 (0.46)	−0.34 (0.35)	−14.1	<0.0001
	B. Acute Coronary Syndrome	0.67 (0.34)			
A vs. C	A. Stroke	0.33 (0.46)	−0.28 (0.35)	−11.1	<0.0001
	C. Heart Failure	0.60 (0.38)			
B vs. C	B. Acute Coronary Syndrome	0.67 (0.34)	0.07 (0.23)	4.3	<0.0001
	C. Heart Failure	0.60 (0.38)			
**Chronic Health States**					
D vs. E	D. Stroke	0.52 (0.38)	−0.30 (0.34)	−12.6	<0.0001
	E. Acute Coronary Syndrome	0.82 (0.17)			
D vs. F	D. Stroke	0.52 (0.38)	−0.05 (0.33)	−2.0	0.0429
	F. Chronic Heart Failure	0.57 (0.32)			
E vs. F	E. Acute Coronary Syndrome	0.82 (0.17)	0.25 (0.28)	13.0	<0.0001
	F. Chronic Heart Failure	0.57 (0.32)			
**Acute vs. Chronic Health States**					
A vs. D	A. Stroke	0.33 (0.46)	−0.20 (0.40)	−7.0	<0.0001
	D. Stroke	0.52 (0.38)			
B vs. E	B. Acute Coronary Syndrome	0.67 (0.34)	−0.15 (0.33)	−6.5	<0.0001
	E. Acute Coronary Syndrome	0.82 (0.17)			
C vs. F	C. Heart Failure	0.60 (0.38)	0.03 (0.31)	1.4	0.1578
	F. Chronic Heart Failure	0.57 (0.32)			

^1^TTO utility scores are on a scale anchored with 0 representing dead and 1 representing full health.

Among health states representing long-term effects of cardiovascular conditions, stroke (D) and chronic heart failure (F) had substantially lower utilities than ACS (E) (difference scores = 0.30 and 0.25, respectively; p < 0.0001). Although the difference between stroke (D) and chronic heart failure (F) was still statistically significant (p = 0.04), the difference between these two health states was of a smaller magnitude (0.05).

Each of the acute health states was also compared directly to its parallel chronic health state. For stroke (A vs. D) and ACS (B vs. E), acute utilities were substantially lower than their parallel chronic utilities (difference = 0.20 and 0.15, respectively; both p < 0.0001). The difference between utilities for acute and chronic heart failure was not significant (0.03; p = 0.16).

### Rates of positive/negative utilities and willingness to trade time

Additional descriptive analyses were conducted to characterize patterns of responses. The majority of participants rated each of the six health states as better than dead (i.e., utility score > 0): A (84.0%); B (96.0%); C (94.5%); D (94.0%); E (100.0%); F (97.0%). Three of the health states (A, D, and F) were rated as equal to dead (i.e., utility = 0) by one participant. The health state receiving the most negative utility ratings was health state A (acute stroke), which was considered worse than dead by 31 (15.5%) participants. Health state E (chronic ACS) did not receive any negative utility scores. The other four health states (B, C, D, F) were rated as negative by 5.5% or less of the sample.

As expected for relatively severe medical conditions, most participants were willing to trade time to avoid living in the acute health states. A total of 97.0% (n = 194) were willing to trade time to avoid stroke (health state A), 84.5% (n = 169) traded time when presented with heart failure (C), and 74.5% (n = 149) traded to avoid ACS (B). Although there was less willingness to trade when presented with the chronic health states, most participants were still willing to trade time to avoid any of the chronic conditions: 87.0% for stroke, 83.0% for chronic heart failure, and 51.0% for ACS.

## Discussion

These results add to previously published utilities for cardiovascular events by differentiating between acute and chronic effects. Estimates of clinically important differences in utilities obtained via direct elicitation methods (e.g., TTO methods) have suggested that differences of 0.05 to 0.10 can be considered important [25,64]. The differences between chronic and acute phases of stroke and ACS identified in the current study clearly exceed these criteria. These findings suggest that it may be important to consider whether utilities represent the acute or chronic impact when selecting scores for use in cardiovascular cost-utility models. In addition, these results raise questions about previously published utilities that have been assessed without knowledge of the duration of time between the cardiovascular event and the utility assessment. Given that the disutility associated with cardiovascular events is likely to vary depending on the duration of time since the event, it is difficult to interpret utility values without this information.

Of the cardiovascular conditions rated in the current study, acute stroke was the least preferred health state with the lowest mean utility and the greatest proportion of negative utility scores (15.5%). Almost all respondents (97.0%) were willing to trade time to avoid living in this health state. Although chronic stroke had a higher utility than acute stroke, the chronic stroke health state was also considered quite aversive by most participants (utility = 0.52). When completing the TTO task, respondents often said the stroke health states were particularly undesirable because of the functional impact likely to be associated with the impairments in cognition and mobility. The acute health state representing a year in which ACS occurs also had a relatively low utility (0.67), while the chronic ACS health state had a notably higher utility, which is consistent with clinicians’ descriptions of the strong recovery often observed in patients experiencing an ACS.

The two heart failure health states also had relatively low utilities, with little difference between acute and chronic heart failure. Unlike stroke and ACS, the chronic heart failure health state was not drafted to represent the long-term impact of acute heart failure. Instead, the acute and chronic health states were qualitatively different from each other, as described by clinicians interviewed for the current study. The acute health state was drafted to describe a patient experiencing an episode of acute heart failure following a previous ACS event (which occurred prior to the year described in the health state), while the chronic episode was drafted to describe chronic heart failure in a patient who had previously experienced an ACS event.

It is challenging to place the utilities for the three acute health states (A, B, C) in the context of previously published utilities for these cardiovascular conditions. Whereas previous results are primarily based on assessments of patient health at either an unknown or known time following a cardiovascular event, the current acute health states represent a year that includes the event. Utilities of the current chronic health states (D, E, F) are more comparable with previously published values, although variation in respondent samples, geographical region, severity of the health state, and utility elicitation methods also limit comparability with previous research. Still, it does appear that utilities in this study are in an expected range based on previously published cardiovascular utilities. For example, a utility of 0.52 representing chronic effects of a moderate stroke (health state D) is roughly in the middle of the range of utilities (0.12 to 0.81) for moderate stroke reported in a review of previous TTO and SG studies [65]. The lower utility score of 0.33 for the year including a stroke (health state A) is similar to relatively low values reported by two previous studies assessing utility with the EQ-5D at time points during the first year after a stroke, ranging from 0.21 to 0.41 [30,32]. In addition, the lower utility for acute stroke than for chronic stroke (difference = 0.20) is consistent with the findings of a recent review, which noted that EQ-5D scores tend to improve over time following a stroke [27]. However, some contrasting results have also been reported, including those from two studies with relatively high utility values during the first year after a stroke [31,34].

When using the current utility scores in a model, researchers would need to be aware of the difference between the acute and chronic health states to ensure that values are being applied appropriately. The three chronic health states (D, E, F) described stable health with a duration of 10 years occurring after an acute event that happened in a previous time period. In contrast, the acute health states (A, B, C) describe an acute event, plus subsequent treatment and recovery in the first year after the acute event. The three acute health states may be conceptualized as *path states* lasting for a total time period of one year. Path states are health states that describe the experience of a hypothetical patient who proceeds through a sequence of different health states [66,67]. Conceptually, the path-state approach is appealing because the states can be designed to represent the typical course of a patient with a particular medical condition. It allows respondents to consider the duration of each state within the sequence when valuing the overall path. However, the limitation of this approach is that the resulting utilities represent the path, rather than the individual events or health state components within the path. If participants are rating the path as a whole, it is not possible to determine the impact of each health-related event within the path. Therefore, when using the utility scores for health states A, B, and C in a cost-utility model, these values would be most appropriately used to represent a one-year cycle in which the cardiovascular event occurs.

Utility assessment based on hypothetical health states is associated with limitations that should be considered when interpreting data. Previous researchers have commented that it would be difficult or impossible to capture the disutility of these events with the EQ-5D or another method of assessing patients’ current health [30,40-42]. Essentially, it is not considered feasible to ask patients to rate their own health during the immediate days or even weeks after a serious cardiovascular event, particularly a stroke, which may cause temporary or permanent cognitive impairment. Therefore, the health state description-based approach was used in the current study because it was feasible to draft and value health state descriptions that included the acute events. Although this method is well-suited for identifying the utility impact of specific medical events, results should be interpreted with appropriate caution. The resulting utility scores represent the specific health states, which are based on clinicians’ descriptions of a typical patient rather than the broad range of symptom severity and outcomes that would be observed in an actual patient sample. Therefore, the extent to which these utilities might differ from values reported by actual patients living in the health states is not known. Still, the utility valuation methodology was selected to maximize comparability to published health state utilities. For example, health states were valued by UK general population participants in a TTO task, which is similar to methods used to value the EQ-5D health states in the MVH study [48,49].

Another possible limitation of the current study is that the acute health states, particularly the description of acute stroke, were longer and more detailed than those used in many similar studies (see health state text in Additional file 1). Based on clinicians’ input during health state development, these details were considered necessary to accurately represent key aspects of a typical year beginning with an event and continuing with a gradual partial recovery. However, due to the length, it is possible that some participants may not have been able to consider all parts of the health states when completing evaluations. Some respondents appeared to attend primarily to aspects of impaired functioning that were particularly salient to them, while possibly overlooking other aspects of the health states. If participants fail to notice and consider aspects of the health states, this would lead to some degree of error and inconsistency in the ratings. Although it is possible that this occurred in some cases, steps were taken to minimize this risk. For example, participants were only asked to rate three acute health states, rather than the larger number of health states often included in other studies, and the three chronic health states were presented separately to avoid providing too much information at once. In addition, the acute health states were formatted in a series of sections with headers indicating the chronological structure, which was intended to ensure that the content and sequence of information would be clear. Finally, interviewers were carefully trained to ask questions to ensure that respondents understood and attended to all aspects of the health states, while avoiding leading questions that could bias valuations.

The sample recruitment strategy should also be acknowledged as a limitation of the current data. Utilities were based on evaluations of a general population sample because most health technology assessment agencies prefer that utilities represent the general public or societal view [59-62]. However, the sample was recruited in only two UK locations, and it should not be considered nationally representative. The extent to which the current utility scores would differ from values derived in a nationally representative sample is not known.

## Conclusions

Despite limitations, this study and its methodology have several strengths. The health state-based method allows for an addition to published post-event utilities because the acute values are based on health states that include cardiovascular events. Thus, the current utilities provide a unique option that may be used to represent the impact of cardiovascular conditions in models, possibly supplementing utilities derived from actual patients with standardized preference-based measures. With the current utilities, cost-utility models comparing treatments that may delay or prevent the onset of cardiovascular events can distinguish between acute and chronic impact of these cardiovascular events.

## Additional file

Additional file 1:
**Cardiovascular Health States.**

## Abbreviations

ACS: Acute coronary syndrome
CUA: Cost-utility analyses
MVH: Measurement and Valuation of Health study
QALY: Quality-adjusted life years
SG: Standard gamble
TTO: Time trade-off
UK: United Kingdom
US: United States
