# Supplementary material for: Acute and chronic impact of cardiovascular events on health state utilities
Source: BMC Health Serv Res. 2015 Apr 22;15:173. doi: 10.1186/s12913-015-0772-9 (PMC4408571; doi:10.1186/s12913-015-0772-9)
Supplement: Additional file 1: — Cardiovascular Health States. [file 12913_2015_772_MOESM1_ESM.docx]

**Additional file: Cardiovascular Health States**

**HEALTH STATE A: STROKE (1-YEAR WITH EVENT)**

1. **Event**

- Early in the year, you develop a blockage in one of the major arteries of your brain so blood can no longer flow through it. As a result, part of your brain is damaged, and you suddenly have the following symptoms:
- Weakness in your arm, leg, and face on one side of your body.
  - - Your arm feels weak and uncoordinated. For example, you have difficulty using a fork to eat
    - Your leg feels weak. You have difficulty walking on your own.
    - You have muscle weakness on one side of your face. This side appears droopy, and you have difficulty smiling.
- You have difficulty swallowing.
- Your speech becomes slurred and unclear. People around you say your speech is hard to understand.
- You feel confused.

1. **Hospital**

- You are quickly taken to hospital. Immediately, several doctors and nurses speak with you, and you begin receiving a range of tests including a blood test, ECG (heart rhythm test), a CT scan of brain tissue, and a scan of arteries in your neck and brain. The scans help doctors see what is happening so they can determine the most appropriate course of treatment.
- You are monitored closely in hospital for the first few days. Then, you remain hospitalized for 3 to 4 weeks for treatment, care, and rehabilitation.
- During rehabilitation, you receive 3 kinds of therapy:
  1. Physiotherapy to help you strengthen muscles (e.g., walking).
  2. Speech therapy to help you speak more clearly and reduce confusion. A speech therapist also helps you re-learn how to swallow and eat without choking.
  3. Occupational therapy to help you cope with your new problems and begin to re-learn skills (e.g., using a fork, writing).
- Because of your difficulty swallowing, you eat softer foods so that you do not choke. You need other people to cut your food for you.

**3. First 6 Months After the Event**

- After returning home, your symptoms gradually improve for the first few months, but you continue to have difficulties:
  - Your speech gradually becomes clearer, but you are frustrated and angry because you have difficulty speaking clearly.
  - You need a walking stick or Zimmer frame to walk.
  - You still have some weakness on one side of your body.
  - You have difficulty remembering things and thinking clearly.
- You take several medications each day including aspirin, a cholesterol lowering tablet, and a blood pressure lowering tablet. You see a doctor about once every 3 months.
- Because of your difficulties, you can no longer work.
- You rely on family and friends to help you with many daily activities, such as getting around and shopping.
- You now have increased responsibility for managing your health. You try to follow your doctor’s recommendations, including:
  - Maintaining a healthy weight.
  - Exercising regularly (although you still have physical limitations).
  - Reducing the amount of fat and salt in your diet.
  - Trying to stop smoking (if you were a smoker).
  1. **7 to 12 Months After the Event**
- Although you have regained many of your abilities, you still have limitations:
  - Your pronunciation is improving, but you still do not speak as clearly as before the event.
  - You can walk by yourself, but you walk more slowly than before the event.
  - You cannot think as clearly as before the event.
  - You have some difficulty remembering things and learning new information.
- You take several medications each day including aspirin, a cholesterol lowering tablet, and a blood pressure lowering tablet. You see a health care provider one or two times during this 6-month period.
- You still have increased responsibility for managing your health. You continue trying to follow your doctor’s recommendations.
- You can no longer work, but you can get around by yourself and perform most daily activities.

**HEALTH STATE B: ACUTE CORONARY SYNDROME (ACS) (1-YEAR WITH EVENT)**

1. **Event**

- Early in the year, you have a sudden blockage in one of the arteries that supplies blood to your heart.
- You have chest discomfort that feels like heaviness, tightness, and a burning feeling. It feels like your chest is being pressed or crushed by a large object.
- You are pale and sweaty.

1. **Hospital**

- You are quickly taken to hospital.  Immediately, several doctors and nurses speak with you, and you begin receiving blood thinners and other medication.  You receive a range of tests including a blood test, ECG (heart rhythm test), and a chest x-ray.
- You are taken to the operating room for a procedure to restore blood flow to your blocked artery. In this procedure, a very small balloon is threaded through an artery from your wrist or groin to the blockage in your heart. This balloon is inflated to open the artery so that blood can flow to your heart, and a very small mesh tube is placed inside the artery to keep it open.
- After the procedure, you are monitored in hospital for about 3 days before going home.

1. **First 6 Months After the Event**

- You participate in a cardiac rehabilitation program in a hospital or other health care setting 2 to 3 days each week, for three months. Each session lasts 1 ½ hours.  The sessions include mild exercise.
- After the rehabilitation program, you are able to continue regular physical activity, but it may be more difficult due to your heart condition.
- You take several medications each day including aspirin, a cholesterol lowering tablet, and a blood pressure lowering tablet.
- You see a doctor 3 times during this 6-month period. In at least one of these visits, you undergo testing to assess your heart.
- You now have increased responsibility for managing your health. You try to follow your doctor’s recommendations, including:
  - Maintaining a healthy weight.
  - Exercising regularly.
  - Reducing the amount of fat and salt in your diet.
  - Limiting stress (e.g., by working at a less stressful job).
  - Trying to stop smoking (if you were a smoker).
  - You worry about the possibility of another heart problem.

1. **7 to 12 Months After the Event**

- You return to the level of physical activity you had before the event.
- You take several medications each day including aspirin, a cholesterol lowering tablet, and a blood pressure lowering tablet.
- You see a doctor 2 times during this 6-month period.
- You still have increased responsibility for managing your health. You continue trying to follow your doctor’s recommendations.
- You sometimes worry about the possibility of another heart problem or a related cardiovascular disease.

**HEALTH STATE C: HEART FAILURE (1-YEAR WITH EVENT)**

1. **Past Event (before the current year)**

- In the past, you had a sudden blockage in one of the arteries supplying blood to your heart.

1. **Current Health**

- You can participate in regular physical activity.
- You take several medications each day including aspirin, a cholesterol lowering tablet, and a blood pressure lowering tablet.
- You see a doctor at least once every year. At these visits, you receive a range of tests including a blood test and a blood pressure test. At some of these visits, you also receive an ECG (heart rhythm test).
- You have increased responsibility for managing your health. You try to follow your doctor’s recommendations, including:
  - Maintaining a healthy weight.
  - Exercising regularly.
  - Maintaining a low-fat and low-salt diet.
  - Limiting stress (e.g., at work).
  - Avoiding smoking.
  - You sometimes worry about the possibility of another heart problem or a related cardiovascular disease.

1. **Event (during the current year)**

- Because of your past heart-related event, your heart is weakened. At some point in the current year, over the course of a week, you gradually develop the following symptoms:
- Weight gain and bloating from excess fluid.
- Shortness of breath that makes you feel like you are suffocating. You feel panicked because you are desperate for breath.
- Swelling in your legs resulting from excess fluid.
- Fatigue.
- Chest pain.
- You are taken to the hospital where several doctors and nurses speak with you. You receive a range of tests including having blood drawn, ECG (heart rhythm test), echocardiogram (ultrasound test to check how well the heart is pumping), and chest x-ray.
- You are given an intravenous (IV) diuretic medication that causes frequent urination to reduce the excess fluid in your body. You are weighed frequently to monitor your fluid retention.
- To improve heart function, you receive medication which controls your heart rate and blood pressure.
- You stay in the hospital for about 7-10 days, where you are monitored and your medications are adjusted.
- You return home after the symptoms (swelling, chest pain, shortness of breath, fatigue) have improved and you have lost fluid weight.
- You worry about the possibility that this could happen again.

**HEALTH STATE D: STROKE (CHRONIC HEALTH STATE, LONG-TERM EFFECTS OF PRIOR STROKE)**

1. **Past Event**

- In the past, you developed a blockage in one of the major arteries of your brain so blood can no longer flow through it. As a result, part of your brain was damaged.

1. **Current Health**

- Although you have regained many of your abilities, you still have limitations.
  - You can pronounce words clearly, but not as clearly as before the event.
  - You can walk by yourself, but you walk more slowly than before the event.
  - You cannot think as clearly as before the event.
  - You have some difficulty remembering things and learning new information.
- You take several medications each day including aspirin, a cholesterol lowering tablet, and a blood pressure lowering tablet. You see a doctor about once every 6-12 months.
- You have increased responsibility for managing your health. You try to follow your doctor’s recommendations, including:
  - Maintaining a healthy weight.
  - Exercising regularly (although you still have physical limitations).
  - Reducing the amount of fat and salt in your diet.
  - Trying to stop smoking (if you were a smoker).
- You can no longer work, but you can get around by yourself and perform most daily activities.

**HEALTH STATE E: ACUTE CORONARY SYNDROME (CHRONIC HEALTH STATE, LONG-TERM EFFECTS OF PRIOR ACS)**

1. **Past Event**

- In the past, you had a sudden blockage in one of the arteries that supplies blood to your heart.

1. **Current Health**

- You can participate in regular physical activity.
- You take several medications each day including aspirin, a cholesterol lowering tablet, and a blood pressure lowering tablet.
- You see a doctor at least once every year. At these visits, you receive a range of tests including a blood test and a blood pressure test. At some of these visits, you also receive an ECG (heart rhythm test).
- You have increased responsibility for managing your health. You try to follow your doctor’s recommendations, including:
  - Maintaining a healthy weight.
  - Exercising regularly.
  - Maintaining a low-fat and low-salt diet.
  - Limiting stress (e.g., at work).
  - Avoiding smoking.
- You sometimes worry about the possibility of another heart problem or a related cardiovascular disease.

**HEALTH STATE F: CHRONIC HEART FAILURE**

1. **Past Event**
   - In the past, you had a sudden blockage in one of the arteries that supplies blood to your heart.
2. **Current Health**

- Because of your past heart-related event, your heart is weakened, and you have persistent symptoms.
  - You feel fatigued, which means you have low energy.  This limits your ability to perform daily activities, such as housework and walking to your car or public transportation.
  - You have shortness of breath when performing mildly strenuous activities, such as walking 25 meters or climbing one flight of stairs. You walk and climb stairs slowly.
  - You have shortness of breath when lying down and you need to use pillows to prop up your head and chest.
- To improve heart function, you take tablets each day to control heart rate and blood pressure. You also take aspirin and a cholesterol-lowering tablet each day.
- Your condition causes you to have extra fluid in your body. Therefore, you take a tablet that causes you to urinate frequently in order to reduce the amount of fluid in your body.  This frequent urination impacts your ability to be active with friends and family.
- You can work part-time, but not full-time
- You see a doctor twice a year.
- You have increased responsibility for managing your health. You try to follow your doctor’s recommendations, including:
  - Maintaining a healthy weight.
  - Exercising regularly (although because of your heart condition, exercise is difficult for you).
  - Maintaining a low-fat and low-salt diet.
  - Avoiding smoking.
  - Limiting how much fluid you drink.
  - Weighing yourself regularly to monitor fluid retention.
